# Whole cell biosynthesis of 1-methyl-3-phenylpropylamine and 2-amino-1,3,4-butanetriol using *Komagataella phaffii* (*Pichia pastoris*) strain BG-10 engineered with a transgene encoding *Chromobacterium violaceum* ω-transaminase

**DOI:** 10.1016/j.heliyon.2019.e02338

**Published:** 2019-08-20

**Authors:** Stephanie Braun-Galleani, Maria-José Henríquez, Darren N. Nesbeth

**Affiliations:** Department of Biochemical Engineering, University College London, Bernard Katz Building, London, WC1E 6BT, United Kingdom

**Keywords:** Bioengineering, Biotechnology, Chemical engineering, *Komagataella phaffii*, *Pichia pastoris*, Transaminase, Whole cell biocatalyst

## Abstract

We have engineered strain BG-10 of the methylotrophic yeast *Komagataella phaffii* for use as an effective whole cell biocatalyst. We introduced into the yeast a transgene encoding a *Chromobacterium violaceum* ω-transaminase for transcription in response to methanol induction. The strain was then assessed with respect to its growth performance and biotransformation of a fed ketoalcohol substrate to an amino-alcohol. In the resultant strain, BG-TAM, methanol induction did not compromise cell growth. Successful bioconversion of fed substrates to the by-product, acetophenone, indicated transaminase activity in shake flask-cultivated BG-TAM cells. We then used bioreactor cultivation to exploit the high levels of biomass achievable by *Komagataella phaffii*. In a 900 μL reaction the BG-TAM strain at OD_600_ = 1024 achieved up to 0.41 mol mol^−1^ (mol_product_ mol_substrate_^−1^) yield on substrate (Yp/s) for production of 1-methyl-3-phenylpropylamine and a space time yield (STY) of 0.29 g L^−1^ h^−1^ for production of 2-amino-1,3,4-butanetriol. We have shown that transamination, an important step for bespoke synthesis of small molecule medicines, is biologically realisable using enzymes with a broad substrate range, such as ω-transaminases, within living yeast cells that are fed low-cost substrates for bioconversion.

## 1 Introduction

The unicellular methylotrophic yeast *Komagataella phaffii (K. phaffii)*, formerly known as *Pichia pastoris* (Kurtzman, 2009), is a well-established platform for heterologous protein production (Byrne, 2015; Macauley-Patrick et al., 2005). The initial genome sequence of *K. phaffii* (De Schutter et al., 2009; Küberl et al., 2011) has been further refined and annotated (Sturmberger et al., 2016), supporting the development of genetic tools for its manipulation (Ahmad et al., 2014; Vogl et al., 2016). *K. phaffii* can grow to very high cell densities, exceeding 150 g L^−1^ of dry cell weight, using inexpensive and simple media and carbon sources. Abad et al. (2010) demonstrated the effectiveness of *K. phaffii* as a whole cell biocatalyst by showing that cells engineered to express *Trigonopsis variabilis* D-amino acid oxidase could be used effectively for D-amino acid oxidation. Wei et al. (2018) engineered *K. phaffii* strain GS115 to overexpress a native transketolase and in whole cell biocatalysis the resultant strain achieved high yields of the chiral sugar L-erythrulose from prochiral substrates. Bea et al. (2010) showed that *K. phaffii* cells engineered to express an ω-transaminase from *Vibrio fluvialis* strain JS17 achieved kinetic resolution of racemic α-methylbenzylamine mixture to an enantiomeric excess of 99% (R)-α-methylbenzylamine.

The ω-transaminase designated CV 2025 (Kaulmann et al., 2007) from *Chromobacterium violaceum* (*C. violaceum*) has shown promising results in the production of chiral amino-alcohols, such as 2-amino-1,3,4-butanetriol (ABT), using bacterial whole cells (Ingram et al., 2007; Rios-Solis et al., 2015). ABT is a key synthon in the production of many small molecule drugs (Ager et al., 1996; Sehl et al., 2015), including protease inhibitors (Kwon and Ko, 2002) and antiretrovirals (Raghavan et al., 2010). Bioconversion using ω-transaminases, either purified (Heintz et al., 2017) or within whole cells (Shin and Kim, 1999; Slabu et al., 2017; Gomm and O'Reilly, 2018) has also been used for production of chiral amines, another class of synthon utilised extensively in synthesis of bioactive natural products, agrochemicals and active pharmaceutical ingredients (Kelly et al., 2018).

In this work, we engineered the *K. phaffii* strain BG-10, available commercially from ATUM, to overexpress the bacterial transaminase CV2025 in the new strain, designated BG-TAM. We established the growth performance of the BG-TAM strain and tested its whole-cell biocatalytic production of both the chiral amino alcohol ABT, and the chiral amine 1-methyl-3-phenylpropylamine (MPPA), when fed low-cost substrates.

## 2 Materials and methods

### 2.1 Strains and reagents

*Komagataella phaffii* strain BG-10 and plasmid pJ902-15 were purchased from ATUM. *K. phaffii* biomass concentration was measured as optical density at 600 nm (OD_600_) using a spectrophotometer. The *C. violaceum* CV2025 ω-transaminase gene (NCBI sequence reference WP_011135573.1) was obtained from GenScript. Zeocin was purchased from Thermo Fischer Scientific.

### 2.2 Construction of the *K. phaffii* BG-TAM strain harbouring the CV2025 transaminase gene

The *C. violaceum* CV2025 gene was codon-optimised for expression in *K. phaffii* using the OptimumGene™ algorithm by GenScript. The optimised coding sequence is available as a public data set (Braun-Galleani et al., 2018). The 1.4 kb CV2025 gene was flanked by *Bsa*I sites enabling subcloning into a lone *Bsa*I site present in pJ902-15 to generate the new, 4.9 kb plasmid, pJ-CV2025. BG-10 cells were transformed using 20 μg of Sac*I*-linearised pJ-CV2025 by electroporation (1500 V, 200 Ω, 25 μF) and subsequently plated onto YPD agar plates supplemented with 1 M sorbitol and 200–1000 μg mL^−1^ of zeocin to select for stable integrants.

Colony PCR was used to confirm the presence of integrated pJ-CV2025 in the *AOX1* locus in zeocin-resistant colonies using the primer, CCAAAGACGAAAGGTTGAATG, which was designed to anneal within the *AOX1* promoter and the primer, GATAATTCGACAACAGCAGG, designed to anneal within the codon-optimised *C. violaceum* CV2025 gene at a position predicted to be 300 base pairs downstream of the *AOX1* promoter only in transformants. Transformant colonies, which were both zeocin resistant and positive by colony PCR, were designated ‘BG-TAM’ and a master cell bank of clones was generated and cryopreserved at -80 °C.

### 2.3 Shake flask cultivation of *K. phaffii*

*K. phaffii* BG-10 and BG-TAM strains were grown for 18 h in 250 mL baffled flasks at 30 °C with 250 rpm to an OD_600_ ∼ 10 in 50 mL of BMGY medium (1% w/v yeast extract, 2% w/v peptone, 100 mM pH 6.0 potassium phosphate buffer, 0.4 μg mL^−1^ biotin, 1% v/v glycerol and 1.34% v/v yeast nitrogen base), a base medium for preparation of minimal and synthetic defined yeast media. Cells were harvested by centrifugation at 5,000 rpm and 4 °C for 5 min, washed in phosphate buffer and resuspended in 50 mL BMMY medium (same constituents as BMGY but substituting 1% v/v glycerol for 2% v/v methanol) to an OD_600_ = 5. 2% v/v methanol was added every 24 h to maintain induction of the *AOX1* promoter. Samples were harvested after 48 h of induction and used for bioconversion measurements.

### 2.4 Bioreactor cultivation of *K. phaffii*

Cultivation in bioreactor was performed using a Multifors 1L device (INFORS HT). The Multifors 1L device has been reported previously to achieve OD_600_ in the range of 800–1000 and DCW of 150–200 g L^−1^ during *K. phaffii cultivation* by Templar et al. (2016) and Wei et al. (2018) respectively. For a starter culture, 150 mL BMGY in a 1 L baffled flask was inoculated with a 1.5 mL cryostock, and incubated at 30 °C and 250 rpm, until an OD_600_ = 60 was reached. This was used as inoculum for 600 mL of basal salt medium (BSM: 1 L with RO water with 26.7 mL 85% w/v H_2_PO_4_, 0.93 g CaSO_4_, 18.2 g K_2_SO_4_, 14.9 g MgSO_4_·7H_2_O, 4.13 g KOH, 40 g glycerol and 12 mL of ‘Pichia Trace Metal 1’ (PTM1) solution. PTM1 had the following constituents: 6.0 g L^−1^ CuSO_4_·5H_2_O, 0.08 g L^−1^ Nal, 3.0 g L^−1^ MnSO_4_·H_2_O, 0.2 g L^−1^ Na_2_MoO4·2H_2_O, 0.02 g L^−1^ H_3_BO_3_, 0.5 g L^−1^ CoCl_2_, 20.0 g L^−1^ ZnCl_2_, 65.0 g L^−1^ FeSO_4_·7H_2_O, 0.2 g L^−1^ biotin and 5.0 mL L^−1^ of 96% v/v H_2_SO_4_. Cultivation parameters and methanol induction conditions followed Invitrogen guidelines for *Pichia* fermentation (Invitrogen, 2002) with cultivation conditions of 30 °C, pH 5.0 and dissolved oxygen kept above 20% by increasing agitation speed or pure oxygen sparging. Briefly, cultivation was performed in batch mode for ∼18 h, until glycerol was completely consumed, and subsequently switched to a glycerol fed-batch regime (50% w/v glycerol feed containing 12 mL L^−1^ PTM1 trace salts, 18.15 mL h^−1^ L^−1^ initial fermentation volume) for 6 h, followed by a methanol fed-batch phase using 100% methanol supplemented with 12 mL L^−1^ PTM1 trace salts at the following feed rates: 3.6 mL h^−1^ L^−1^ for 1 h (M1), 7.3 mL h^−1^ L^−1^ for 2 h (M2), and final feed rate of 10.9 mL h^−1^ L^−1^, which was maintained for 48 h. Samples for growth rate monitoring were harvested at t = 0 and t = end of each feeding phase. The measurement of dry cell weight was carried out by putting the cell pellet of 1 mL of culture in an oven (100 °C) and measuring weight in an analytical balance every 24 h until constant (∼96 h). Samples for bioconversions were harvested after the initial batch stage and every 24 h after methanol induction, the supernatant removed and the cell pellet stored at -20 °C.

### 2.5 Bioconversion reactions

All bioconversion reactions were carried out in 900 μL final volume using whole cells at 30 °C and 500 rpm in a thermomixer (Eppendorf ThermoMixer® C). Pelleted shake flask and bioreactor samples were thawed and re-suspended to 600 μL in a pH 7.5 solution of 0.2 mM pyridoxal phosphate (PLP) in 200 mM, 4-(2-hydroxyethyl)-1-piperazineethanesulfonate (HEPES) buffer to allow the PLP cofactor to associate with transaminase within cells. These cell suspensions were then incubated in 1.5 mL glass vials for 20 m, followed by the addition of 300 μL of substrate solution, consisting of 200 mM HEPES buffer pH 7.5, 0.2 mM pyridoxal phosphate and substrate levels needed to provide the final concentrations indicated in the Results section. Aliquots of 100 μL were taken from the bioconversion reactions at different time points, centrifuged at 12,000 rpm for 10 m at 4 °C, before the supernatant was removed, retained and stored at -20 °C for further analysis.

### 2.6 Measuring substrates and products

An integrated Dionex Ultimate 3000 HPLC system fitted with an ACE 5 C18 reverse phase column (150 mm × 4.6 mm, 5 μm particle size) controlled by Chromeleon 7 software (Thermo Fisher Scientific) was used to quantify MBA, ACP, PB, MPPA and ABT. Samples were quenched and diluted with 0.1% v/v TFA prior to analysis. To analyse MBA and ACP, two mobile phases were used: 100% acetonitrile (A) and 0.1% (v/v) trifluoroacetic acid (B). A gradient was run from 15% A/85% B to 72% A/28% B over 9 min, followed by a re-equilibration step for 2 m (oven temperature 30 °C, flow rate 1 mL/min). UV detection was carried out at 260 nm, with detection times of 3.6 m for MBA, and 7.8 m for ACP.

For MPPA and PB analysis, two mobile phases were used: 0.1% (v/v) trifluoroacetic acid (C) and 0.095% trifluoroacetic acid/80% acetonitrile in MilliQ water (D). A gradient of 40% C/60% D to 100% D was run over 15 min, followed by 2.5 m wash with 100% D, and a subsequent re-equilibration step for 2.5 m (oven temperature 30 °C, flow rate 1 mL/min). UV detection was carried out at 254 nm, with retention times of 2.7 m for MPPA, and 7.7 m for PB.

For ABT measurement, 6-aminoquinolyl-n-hydroxysuccinimidyl carbamate (AQC) was used as derivatization agent. AQC, provided by Dr Fabiana Subrizi from UCL Department of Chemistry, was solubilized in dry acetonitrile (10 mg mL^−1^) and stored in dry conditions for no longer than 2 weeks. Typically, a volume of 75 μL of AQC solution was added to 150 μL of bioconversion supernatant previously diluted in 0.02 M borate buffer pH 8.8, reaching a final volume of 225 uL. This mixture was centrifuged prior to analysis. To analyse ABT, two mobile phases were used: 140 mM sodium acetate, pH 5.05 (E) and 100% acetonitrile (F). A gradient of 85% E/15% F to 100% F over 10 m (flow rate 0.5 mL min^−1^), followed by a 2.5 m wash with 40% E/60% F and a 2.5 m wash with 100% E (flow rate 1 mL min^−1^). A re-equilibration step was run for 5 min. UV detection was carried out at 254 nm, with a retention time of 6.1 min. For quantification of ERY, an Aminex HPX-87H reverse phase column (300 × 7.8 mm, Bio-Rad, USA) was used. The system was run at 60 °C with 0.6 mL min^−1^ isocratic flow of 0.1% trifluoroacetic acid as mobile phase. UV Detection was carried out at 210 nm with a retention time of 11.5 min. The molar extinction coefficient (ε) at wavelength of 260 nm was determined to be 4878.05 M^−1^ cm^−1^ for MBA and 4405.29 M^−1^ cm^−1^ for ACP. At 254 nm ε was determined to be 171.32 M^−1^ cm^−1^ for PB, 168.86 M^−1^ cm^−1^ for MPPA and 2890.17 M^−1^ cm^−1^ for derivatised ABT.

## 3 Results and discussion

### 3.1 Growth and methanol tolerance of BG-TAM strain unchanged from parental strain

**Fig. 1:**
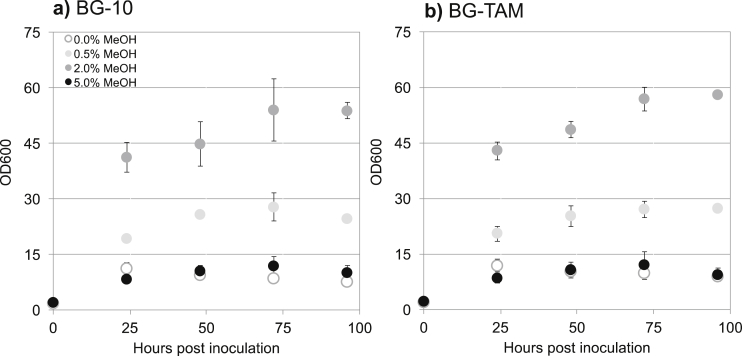
Wildtype BG-10 strain growth performance maintained in BG-TAM strain engineered for overexpression of CV20205 transaminase. Cell growth profile for *K. phaffii* strains: BG-10 (a) and transformant cell line BG-TAM (b) grown in buffered minimal medium supplemented with different methanol concentrations (as indicated in figure key) in 250 mL shake flasks incubated at 30 °C and 250 rpm. Results are an average of n = 2 cultivations, error bars indicate standard error.

We tested whether transaminase overexpression impacted the growth characteristics of *K. phaffii* strain BG-TAM, given that endogenous transaminases form part of the native metabolic networks of the organism. BG-TAM and BG-10 strains were each cultivated in the presence of up to 5% v/v methanol. The BG-TAM growth profile (Fig. 1a) did not deviate markedly from that of the parental strain BG-10 (Fig. 1b). The highest concentration of 5% v/v methanol suppressed growth in both strains, whereas 2% v/v provided the best results for cell density and growth rate. This methanol concentration was used subsequently for induction in the shake flask experiments of this study.

### 3.2 Indication of whole cell transaminase activity in BG-TAM cells cultivated in shake flasks

**Fig. 2:**
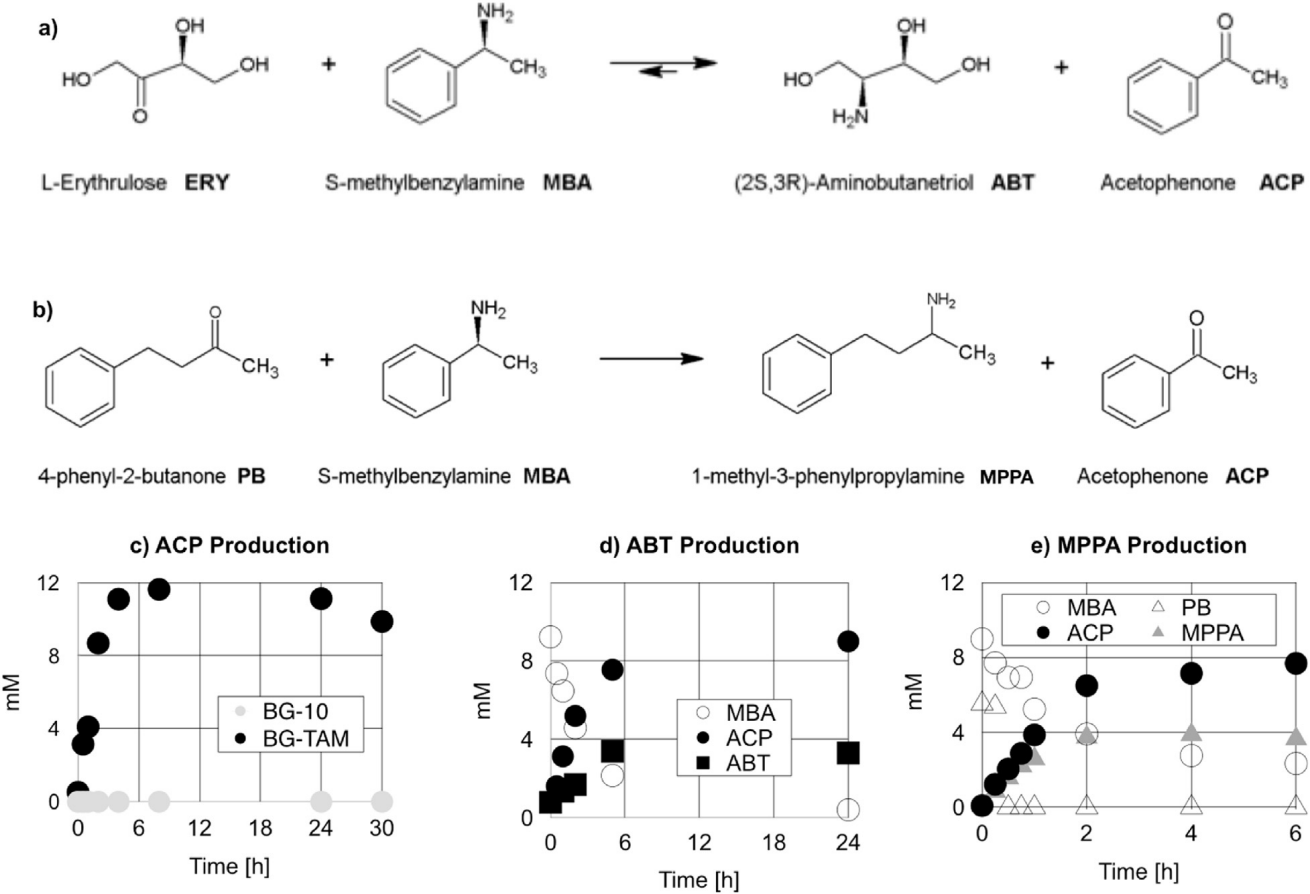
Transaminase activity in BG-TAM whole cells cultivated in shake flasks. Reaction schemes detail the synthesis of ABT (a) and MPPA (b). Level of ACP generated by strains BG-10 and BG-TAM fed 10 mM MBA and 30 mM ERY as substrates (c). Production of ABT by strain BG-TAM fed 10 mM MBA and 30 mM ERY (d). Production of MPPA by strain BG-TAM fed 10 mM MBA and 10 mM PB (e). Cell concentration normalised to OD_600_ = 39 in all assays. The symbol keys for the graphs obstruct no data points.

BG-10 and BG-TAM cells were cultivated in shake flasks in the presence of 2% v/v methanol for 48 h. Harvested cells were diluted or concentrated to a normalised concentration of OD_600_ = 39, for purposes of comparison, and incubated with substrates to assess whole cell biocatalytic activity for production of ABT and MPPA by the pathways illustrated in Fig. 2a and b, respectively. When cells of the BG-TAM were incubated with 10 mM MBA and 30 mM ERY, production of ACP was observed, whereas under the same conditions cells of the parental BG-10 strain resulted in no ACP production (Fig. 2c). We interpreted this observation as further evidence that the plasmid pJ-CV2025 had integrated at the AOX1 locus as intended and that expression of the codon-optimised CV2025 gene was responsible for the observed ACP accumulation. Shake flask-cultivated BG-TAM cells were prepared as above for bioconversion of MBA and ERY to ABT and also for bioconversion of MBA and PB to MPPA. Both bioconversions showed activity, with production of around 4mM product for both ABT (Fig. 2d) and MPPA (Fig. 2e).

### 3.3 Cultivation of BG-TAM strain to high cell density in a 1L bioreactor

**Fig. 3:**
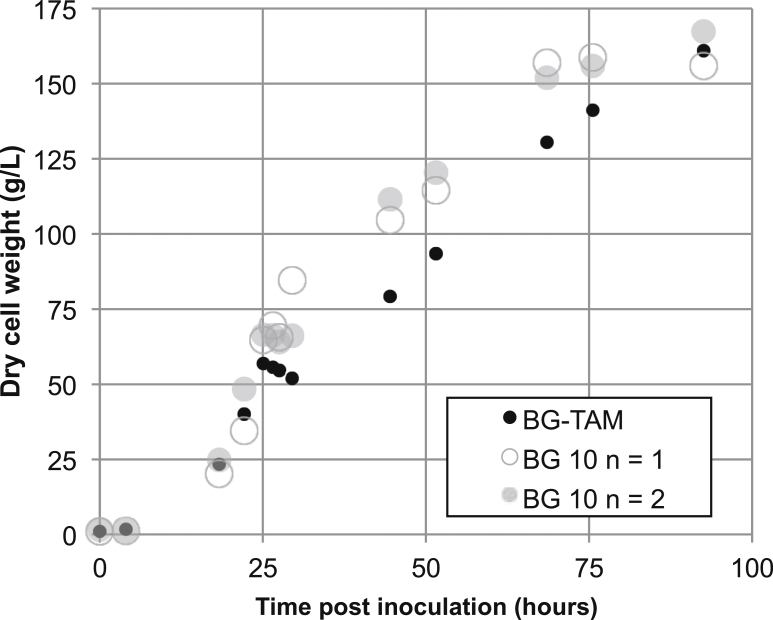
Bioreactor cultivation of BG-TAM to high cell density. Engineered BG-TAM and parental BG-10 strains were cultivated in parallel using a Multifors 1 L bioreactor system. A standard Invitrogen fermentation protocol was used in which an initial glycerol batch phase was applied until 18.5 h post-inoculation, followed by glycerol fed-batch growth until approximately 26 h post-inoculation. From 26.5 h post-inoculation onward methanol fed-batch growth was applied.

BG-TAM and parental BG-10 strain cells were cultivated in a 1L bioreactor comprising firstly a glycerol batch and fed-batch feeding to achieve high biomass, followed by fed-batch methanol feeding to induce the *AOX1* promoter that controls expression of the transgene. Under this cultivation regime, the unmodified parental BG-10 strain and the engineered BG-TAM strain achieved cell densities of OD_600_ ∼ 900–1000, after 48 h of methanol induction. The growth profile illustrated in Fig. 3 shows cell biomass accumulation data from three cultivations performed in parallel using the Multifors 1L bioreactor system. Growth of the engineered BG-TAM strain was comparable to that of the unmodified strain, with matching levels of final biomass achieved at harvest. Given that the strains also showed similar growth performance during shake flask cultivation (Fig. 1), under methanol induction, we concluded that overexpression of the CV20205 transaminase gene in BG-TAM did not considerably perturb viability of the BG-TAM strain relative to the parental strain.

### 3.4 High density whole cell bioconversion as a function of substrate concentration

**Fig. 4:**
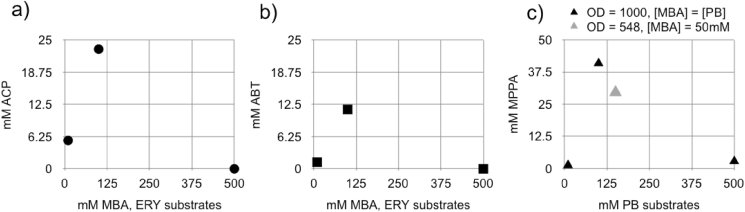
Upper limits of substrate concentration for BG-TAM whole cell biocatalysts at high cell density. BG-TAM was cultivated in a bioreactor to high cell density (OD_600_ = 1024) and biocatalytic performance measured as a function of substrate concentration. Graphs show bioconversion of the fed substrate pair MBA/ERY to ACP (a, black circles) and ABT (b, black squares) and bioconversion of MBA/PB to MPPA (c, triangles – black and grey to indicate different cell densities and substrate concentrations used in the reaction).

Bioreactor-cultivated BG-TAM cells were harvested after 66 h of methanol induction (92.5 h post inoculation) when an OD_600_ = 1024 was achieved (final time point, Fig. 3). We then mapped the limits of substrate concentration that high cell density suspensions of BG-TAM cells could tolerate whilst maintaining bioconversion. We measured bioconversion achieved by cells incubated with 10–30 mM substrate, the concentrations we had used for shake flask samples, and then increased substrate concentration to 100 mM and finally to 0.5 M (Fig. 4).

A concentration of 0.5 M MBA and ERY abolished production of both ACP (Fig. 4a) and ABT (Fig. 4b), establishing an upper limit on the performance of the whole cells for this reaction. By contrast, the whole cell biocatalyst retained a level of activity in 0.5 M substrates PB and MBA, producing 2.82 mM MPPA. We concluded from these observations that the upper limit for substrate tolerance for the ABT reaction is not due solely to general stress of the enzyme or living cells but is in part substrate-specific. At a concentration of 100 mM, all substrates were well-tolerated and resulted in the highest observed bioconversion levels of 11.48 mM for ABT, and 29.6 mM for MPPA (Fig. 4). The data in Fig. 4 suggest future investigations would be warranted to map biocatalysis performance for substrate concentrations between 0.1 M and 0.5 M.

## 4 Conclusions

Schrewe et al. (2013) proposed a battery of performance measures best suited to whole cell biocatalysts, which could be useful for researchers or industrialists across a range of different process configurations and needs. In Table 1 we collated the performance metrics for BG-10 strain whole cells used in the three best-performing conditions, with respect to product concentration, observed in Fig. 4. Table 1 shows that BG-10 whole cells achieved a best yield on substrate (Yp/s) of 0.41 mol mol^−1^ (mol_product_ mol_substrate_^−1^), which is comparable to the Yp/s of 0.52 mol mol^−1^ reported by Wei et al. (2018) for whole cell biocatalysis using a *K. phaffii* strain engineered to overexpress transketolase, an enzyme type that typically exhibits high activity in bioconversions. As ω-transaminases have been shown to be responsive to mutational approaches to increase their performance (Deszcz et al., 2015), the performance data reported here with an unmodified ω-transaminase certainly encourages further investigation of whole cell applications.

**Table 1:** Schrewe metrics for whole cell biocatalysis using strain BG-TAM. Performance data gathered using post induction samples of *K. phaffii* BG-TAM cultivated in a 1 L bioreactor. The best-performing reaction plotted in Fig. 4b, in which 11.48 mM ABT was measured, was analysed further here in the column ABT-X. The reaction plotted in Fig. 4c, in which 40.9 mM MPPA was measured, was analysed further here in the column MPPA-X. The reaction plotted in Fig. 4c, in which 29.6 mM MPPA was measured, was analysed further here in the column MPPA-Y.

Reaction values	*ABT-X*	*MPPA-X*	*MPPA-Y*
Starting [MBA]/[PB]		100 mM/100 mM	50 mM/150 mM
Starting [MBA]/[ERY]	100 mM/100 mM		
[ABT] after 4 h reaction MW 121.135	11.48 mM 1.4 g L^−1^		
μM ABT per min	47.8 μM min^−1^		
[MPPA] after 4 h reaction MW 149.23		40.9 mM 6.1 g L^−1^	
[MPPA] after 2 h reaction			29.6 mM 4.4 g L^−1^
μM MPPA per min		170.4 μM min^−1^	246.7 μM min^−1^
g/L DCW in 600 μL sample	237.6	237.6	133.75
g/L DCW in 900 μL reaction	160.95	160.95	93.45
**Schrewe metrics**			
STY (g_product_ L^−1^ hr^−1^)	0.35 g L^−1^ hr^−1^	1.52 g L^−1^ hr^−1^	2.2 g L^−1^ hr^−1^
Specific activity (U g_CDW_^−1^)	0.30 U g_CDW_^−1^	1.06 U g_CDW_^−1^	2.64 U g_CDW_^−1^
(U = μmole min^−1^)			
Y_p/s_ (mol_product_ mol_substrate_^−1^)	0.11 mol mol^−1^	0.41 mol mol^−1^	0.30 mol mol^−1^
Y_p/x_ (g_product_ g_CDW_^−1^)	8.7 mg g_CDW_^−1^	37.9 mg g_CDW_^−1^	47.1 mg g_CDW_^−1^

## Declarations

### Author contribution statement

Stephanie Braun-Galleani: Conceived and designed the experiments; Performed the experiments; Analyzed and interpreted the data; Contributed reagents, materials, analysis tools or data; Wrote the paper.

Maria-José Henríquez: Contributed reagents, materials, analysis tools or data.

Darren N. Nesbeth: Conceived and designed the experiments; Analyzed and interpreted the data; Contributed reagents, materials, analysis tools or data; Wrote the paper.

### Funding statement

This work was supported by the UK BBSRC grant BB/M004880/1 within the ERA-NET IPCRES consortium, and CONICYT-Becas Chile (Folio no. 72120390).

### Competing interest statement

The authors declare no conflict of interest.

### Additional information

No additional information is available for this paper.
